# Experimental and Numerical Characterization of High Damping Martensitic CuAlMn Sheets

**DOI:** 10.3390/ma13030529

**Published:** 2020-01-22

**Authors:** Pouya Haghdoust, Antonietta Lo Conte, Simone Cinquemani, Nora Lecis

**Affiliations:** Politecnico di Milano, Department of Mechanical Engineering, Via La Masa 1, I-20154 Milan, Italy; antonietta.loconte@polimi.it (A.L.C.); simone.cinquemani@polimi.it (S.C.); nora.lecis@polimi.it (N.L.)

**Keywords:** SMA, damping, nonlinear

## Abstract

This paper deals with the experimental and numerical characterization of a high damping CuAlMn sheet with a martensitic micro-structure at ambient temperature. A Cu-Al-Mn shape memory alloy containing 11.65 wt.% of Al and 3 wt.% of Mn, was cast and hot rolled to the thickness of 0.4–0.3 mm. Transformation temperatures, micro-structure and mechanical properties were studied. Effects of the heat treatment on damping were investigated, identifying the proper heat treatment to obtain a higher damping. Having to model the amplitude dependent damping of the material investigated, a material model was developed based of cyclic behavior under traction-compression load. The model was validated with experiments on the non-linear damping of the material.

## 1. Introduction

The industry’s demand for materials with a high damping capacity, ranging from the automotive to the aerospace and structural sectors, has led researchers and engineers to focus on the issue. Among the different materials available to achieve damping, the use of shape memory alloys (SMAs) has gained great relevance given their high damping levels due to their unique microstructures [1,2,3]. The damping of SMAs can be associated to two different mechanisms: pseudo-elasticity and dissipation in the martensitic state. The former occurs in SMA materials with an austenetic phase at their operating regime, where the stress induced martensite generates a pseudo-elastic behavior of the material. The latter occurs in SMAs with a martensitic micro-structure at an operating regime, due to the high density of mobile twins and mobile interfaces [4]. While there are several studies in the literature that investigate damping on the basis of a pseudo-elastic behavior of SMAs [3,4], damping in the martensitic state has received less attention, although there are many practical advantages [4,5,6].

Among the SMA systems available, Cu-based alloys are an important group, as they are easy to produce through conventional techniques, and consequently they are economically convenient compared to Nitinol [7]. Moreover, for applications where the desired property is the damping capacity, said group may be a better choice over nickel titanium systems. The highly ordered Cu-based SMAs, such as Cu-Al-Ni and Cu-Zn-Al, are too brittle to be cold worked, while the Cu-Al-Mn alloys, with low aluminum contents, show an excellent ductility, since their parent phase, with an $L_{21}$ structure, possesses a lower degree of order [8].

Cu-Al-Mn systems have already been investigated in a number of studies. In fact, the effects of rolling and heat treatment on the micro-structure and super-elasticity of Cu${}_{71}$Al${}_{18}$Mn${}_{11}$ were investigated by Ji-Li Liu, allowing him to observe a good workability and a good super-elasticity through rolling [9]. Sutou and Kainuma [10] investigated the effects of alloying elements on transformation temperatures, ductility and shape memory properties by using DSC, cold-rolling and tensile test techniques. In this work, the cold-workability was decreased by adding elements such as Ti, Cr, Co, Si and Sn, while the shape memory effect was improved by adding elements such as Ti, Cr, Fe, Co, Ni, Au and Zn. Mallik and Sampath [11] found that by increasing the amount of aluminum, the martensite’s morphology and transformation temperatures changed, producing a negative effect on the super-elasticity. At the same time, they found that an increase in the amount of manganese stabilizes the martensite and improves the super-elasticity of alloys. In other studies [12,13], the same authors compared different compositions of the Cu-Al-Mn system, concluding that the damping capacity of alloys increases when increasing the aluminum content, and when keeping the Cu/Mn ratio constant. They also observed that the damping capacity decreases with an increase in the manganese content, when the amount of aluminum is more or less constant. Finally, they observed a significant decrease in damping in these alloys with aging, which was attributed to a restricted interfacial movement of martensite variants, martensite/austenite and twin boundaries. Lastly, Sutou et al. [14] investigated the effects of the grain size and texture on the damping properties of CuAlMn-based alloys, observing an increase in the damping properties when increasing the relative grain size.

The scope of this study was to produce high damping thin sheets of SMA to be embedded in GFRP (glass fiber reinforced polymer) hybrid composite, as a passive strategy to control the vibrations of the composites’ structures. This hybridization concept has already been investigated by using martensitic Ni${}_{40}$Ti${}_{50}$Cu${}_{10}$ and Cu${}_{66}$Zn${}_{24}$Al${}_{10}$ as high damping materials [15]. However, the former is very expensive and the latter is not ductile enough to be laminated in thin sheets, as is required by the target application. To overcome those problems, the Cu${}_{85.33}$Al${}_{11.65}$Mn${}_{3}$ composition, which exhibits higher damping levels compared to other similar compositions, was investigated for the hybridization of composites [12,13]. Studies were also conducted on the possibility to fabricate thin sheets (with a thickness amounting to 0.3–0.4 mm) according to the thickness required by the hybridization technique. The micro-structures and transformation temperatures of the rolled samples were assessed to guarantee the martensitic microstructure at ambient temperature, selected as the service temperature. The effects of various heat treatments and the grain size on the damping of the system, were studied with the aim of finding a suitable situation for damping.

Furthermore, a numerical model was developed to replicate the exact behavior of the investigated high damping Cu${}_{85.35}$Al${}_{11.65}$Mn${}_{3}$ alloy. Since in the case of high damping materials, non-linearities can not be neglected, classical viscous damping models could not be used in the numerical analysis. The model developed is a modified Masing model, finalized to reproduce the strain dependence of the material internal damping, on the basis of experimental hysteresis loops at different maximum strain amplitudes. The model developed was validated by experimental results and implemented in a user subroutine of the FE Abaqus code, to be used in a numerical simulation of the material behavior.

## 2. Materials and Experiments

### 2.1. Alloy Preparation and Fabrication of Thin Sheets

The original ingot of the investigated material was cast in the shape of a cylinder with a cross section diameter equal to 30 mm and a length of 150 mm. High purity powder was melted together with a nominal composition of Cu${}_{85.35}$Al${}_{11.65}$Mn${}_{3}$ in an induction furnace under an argon atmosphere (The ingot was provided by SAES Getters S.p.A. Lainate (Milano)—Italy.) The selected composition exhibited higher damping compared to other similar compositions selected in previous studies [12,13].

**Figure 1:**
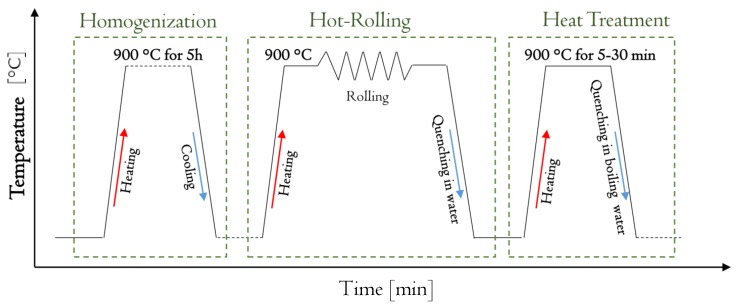
Process for the fabrication of the thin sheets.

The ingot was cut by an abrasive cutting machine into smaller cylindrical samples, which were used to fabricate the thin sheets. The procedure for fabricating the thin sheets from each sample is depicted in Figure 1. The first step of the process consisted in the homogenization at 900 ${}^{{}^{\circ}}$C for 6 h under argon atmosphere to ensure the homogenized chemical composition of each sample. This was followed by a hot-rolling (HR) step. In said step, for each hot-rolling passage, the samples were initially heated at 900 ${}^{{}^{\circ}}$C in furnace and maintained for 5 min; then each was removed from the furnace and quickly put inside the two-roller rolling mill (the roller diameter amounted to 350 mm), followed by rapid water quenching (WQ). This was done until the thickness of the sheets reached 0.3–0.4 mm according to the thickness required in order for the sheets to be embedded in the GFRP composite. The last step of the procedure, as presented in Figure 1, was the heat treatment. In this step, in order to obtain the thermoelastic martensite microstructure, the hot-rolling process was followed by betatizing sample at 900 ${}^{{}^{\circ}}$C with a subsequent quenching in boiling water. The mentioned heat treatment was performed at different times in order to study how it affects the damping of the fabricated sheets.

**Figure 2:**
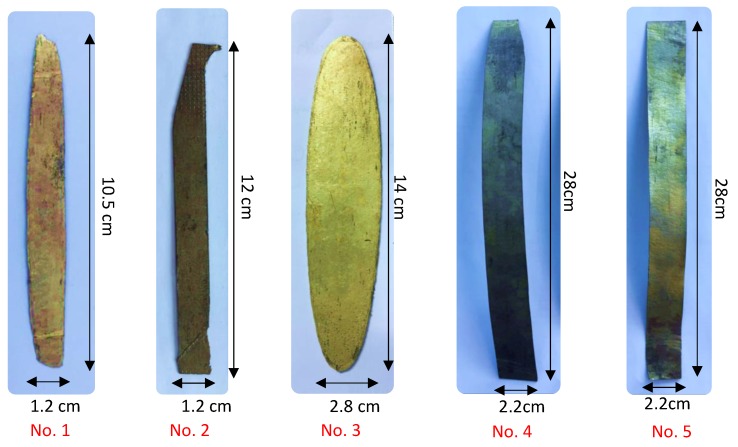
Final shapes of the fabricated sheets.

Details and dimensions of the fabricated sheets are reported in Table 1 and Figure 2. All specimens, except Specimen 3, were hot rolled to thickness in the range of 0.35–0.45 mm. Specimen 3 was hot rolled at 1.1 mm, since it was used for tension-compression tests. With regard to Specimens 1, 2 and 3, only one heat treatment was investigated, while Specimens 4 and 5 were examined with different heat treatments. The thickness of the rolling samples before and after each rolling passage was carefully measured using a digital micrometer and the reduction was calculated according to:

(1) $$\epsilon =\left(h_{0}-h_{1}\right)/h_{0}\times 100\%$$

where $h_{0}$ and $h_{1}$ are the thickness of the sheet samples before and after rolling, respectively. The highest thickness reduction in each rolling passage was equal to 18% with a accumulative reduction of 94.2%.

**Figure 3:**
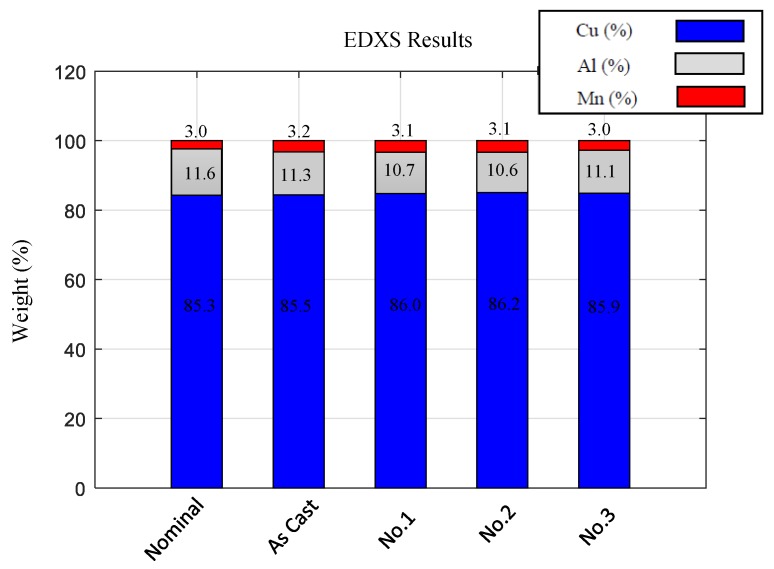
EDXS results of the as-cast and heat treated samples.

The composition of the alloy was checked using an EDXS analysis for the as-cast and the heat treated samples (1, 2 and 3) in order to control the composition. The analyses confirmed that the composition does not vary during the performed procedure. The results are presented in Figure 3. The nominal composition is also presented as reference.

### 2.2. Microstructure and Composition Studies

**Figure 4:**
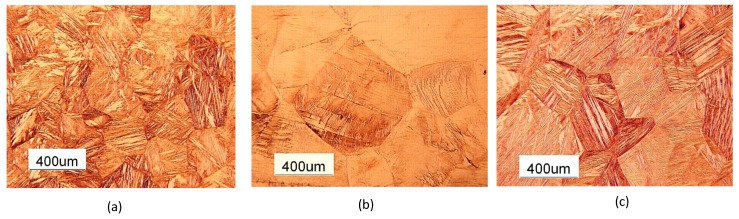
Comparison of the microstructures of Specimen 1. (**a**) As-cast. (**b**) As-rolled. (**c**) Heat treated.

The microstructure of the material, at the different stages of the preparation of the thin sheets and the evolution of the morphology of the martensitic grains and subgrains with heat treatment, were studied using an optical microscope (Leica DFC290) and SEM (ZEISS EVO). Each sample was polished and etched in a solution of FeCl3 (10 g) + HCl (25 mL) + H2O (100 mL) for 5 s. Figure 4 shows the micro-structures of Specimen 1 in as-cast, hot rolled and heat treated conditions respectively. The martensite microstructure in the as-cast situation was transformed into austenite in hot-rolled samples (Figure 4a,b), while a martensitic micro-structure was obtained after the heat treatment (Figure 4c). This microstructure evolution was confirmed for Specimens 2 and 3 after the same heat treatment.

**Figure 5:**
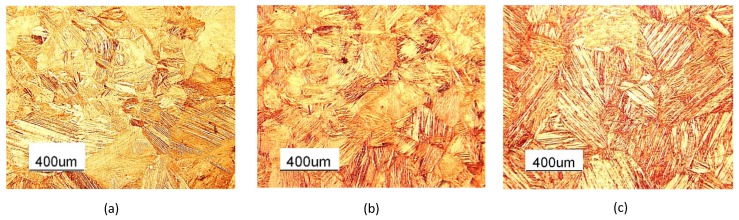
Comparison of the microstructures of Specimen 4 with different heat treatments: (**a**) 7${}^{\prime }$ HT. (**b**) 15${}^{\prime }$ HT. (**c**) 30${}^{\prime }$ HT.

**Figure 6:**
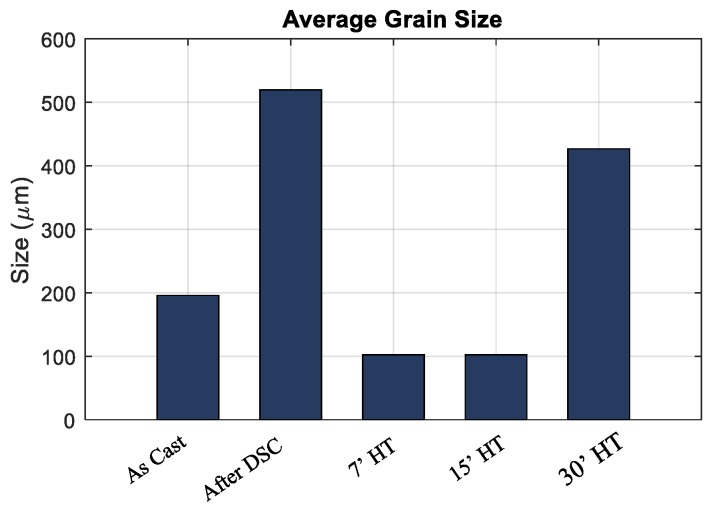
Comparison of the grain sizes for the as-cast, after DSC and different heat treatments.

The comparison of the martensitic grains and their morphologies is presented in Figure 5 for samples with different heat treatment times (Specimen 4 with 7${}^{\prime }$, 15${}^{\prime }$ and 30${}^{\prime }$ heat treatments). It is possible to observe that the morphology of the martensitic grains tends to became more homogeneous, and at the same time, the grain size increases when increasing the heat treatment time. Figure 6 shows the quantitative comparison of the average grain size for the as-cast samples and for samples after the heat treatment, as reported in Table 1, where the lineal intercept method, as introduced in the ASTM E112-12 standard, was used to perform the measurements. The values of different samples in the same conditions were grouped together. The average grain size of a sample after performing DSC was also included in the results as reference value. As mentioned, among the heat treated samples, the one with 30${}^{\prime }$ heat treatment showed the largest grain sizes, nearly two times bigger than the grain size in the case of the as-cast sample. The largest grain sizes were observed in the sample on which a DSC analysis was performed.

**Figure 7:**
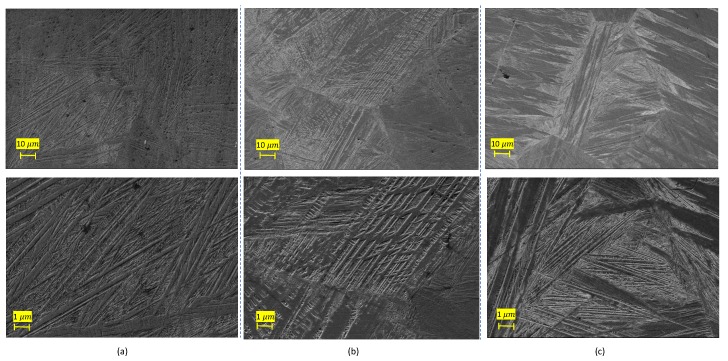
Comparison of the microstructures of the heat treated samples. (**a**) Specimen 4 with 7${}^{\prime }$ HT. (**b**) Specimen 5 with 15${}^{\prime }$ HT. (**c**) Specimen 1 with 30${}^{\prime }$ HT.

To investigate the structures of the martensite grains and subgrains, SEM analyses were performed. As evident in Figure 7, the heat treated Cu${}_{85.35}$Al${}_{11.65}$Mn${}_{3}$ shows the microstructure of samples with heat treatments in increasing order of time. As observed, the heat treated Cu${}_{85.35}$Al${}_{11.65}$Mn${}_{3}$ was mainly composed of martensite plates and lathes. With regard to the sample with a 15′ heat treatment (Figure 7b) a lath type martensite was mainly observed, while in the other two samples martensitic plates were the dominant microstructure. Considering Figure 7a,c, it is possible to conclude that by increasing the heat treatment time from 7${}^{\prime }$ to 30${}^{\prime }$, a decrease in the width of the martensite plate is observed. According to previously performed investigations [14,16], decreasing the martensitic plate width may give rise to a mobile interfaces, which rules the expected high internal damping of the material.

### 2.3. Transformation Temperatures

**Figure 8:**
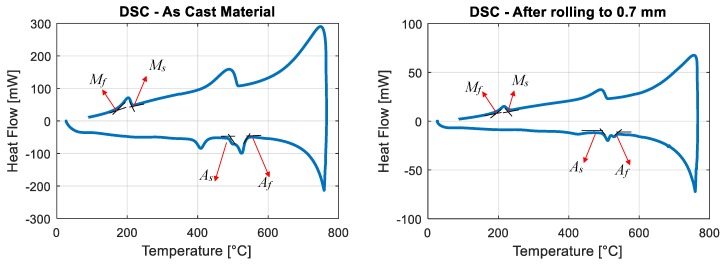
DSC diagram of the as-cast sample and Sample 2 (rolled to 0.7 mm).

The transformation temperatures were determined using a differential scanning calorimeter (DSC). The analysis was performed in the range of 20–750 ${}^{{}^{\circ}}$C by heating/cooling the samples at the rate of 10 ${}^{{}^{\circ}}$C/min. The as-cast sample, the hot-rolled sample (Specimen 2 before reaching to final thickness) and the heat treated sheet (Specimen 2 with a 30${}^{\prime }$ heat treatment) were investigated. The DSC diagrams of the as-cast and hot rolled samples are presented in Figure 8. The DSC diagram of the heat treated sample is not presented, since the samples were to small due to the low thickness of the sheets, and the peaks were not visible to the naked eye. The transformation temperatures of all investigated samples are reported in Table 2. In all cases, a very high martensite-austenite transformation temperature was observed, guaranteeing the martensitic phase at ambient temperature, and the transformation temperatures did not vary for samples with different heat treatments. In each case, the existence of two peaks, both in heating and cooling directions, was considered to be as result of the existing secondary phase, as observed in some cases for Nitinol [17].

### 2.4. Internal Damping

**Figure 9:**
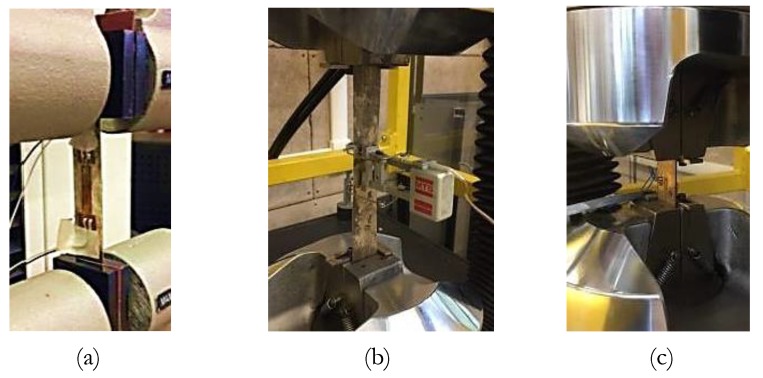
Experimental setups. (**a**) Specimen 1 with two strain gauges on one side of the specimen; (**b**) Specimen 4 with extensometer; (**c**) Specimen 3 with strain gauges on the opposite sides of the specimen.

**Figure 10:**
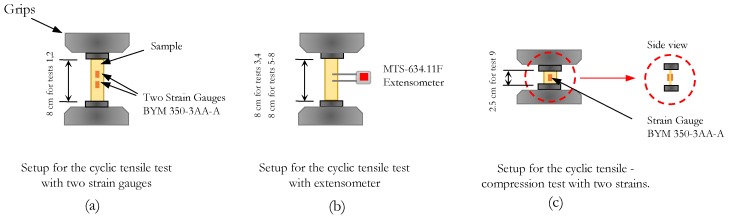
Schematic of the experimental setups. (**a**) Specimen 1 with two strain gauges on one side of the specimen; (**b**) Specimen 4 with extensometer; (**c**) Specimen 3 with strain gauges on the opposite sides of the specimen.

The internal damping of the material at different amplitudes was investigated for different fabricated sheets through experimental measurements of the loss factor parameter. Therefore, cyclic tests were conducted on fabricated sheets using an MTS RT/100 hydraulic machine and three different setups in order to measure the strain on the gauge length of the specimen. The first setup (Figure 9a) was dedicated to Specimen 1 that had a thickness of 0.35 mm. Two BYM 350-3AA-As with a 3.5 mm gauge length were applied on one side of the specimen gauge length, as shown in Figure 10a). The second setup (Figure 9b) was dedicated to Specimens 2, 4, and 5 that had a thickness of 0.35, 0.42, and 0.45 mm respectively. In this case the MTS-634.11F extensometer with a 25 mm gauge length, was mounted on the specimen gauge length (Figure 10b). The third setup (Figure 9c) was dedicated to Specimen 3, with a thickness of 1.11 mm. In this case one BYM 350-3AA-A strain gauge was applied symmetrically on both sides of the specimen (Figure 10c), in order to control and avoid the buckling of the sample in compression.

**Figure 11:**
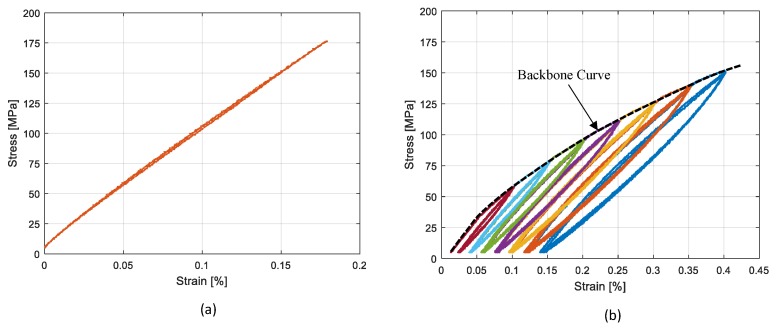
Tensile test results on Specimen 1: (**a**) rolled sheet; (**b**) 30${}^{\prime }$ HT sheet.

**Figure 12:**
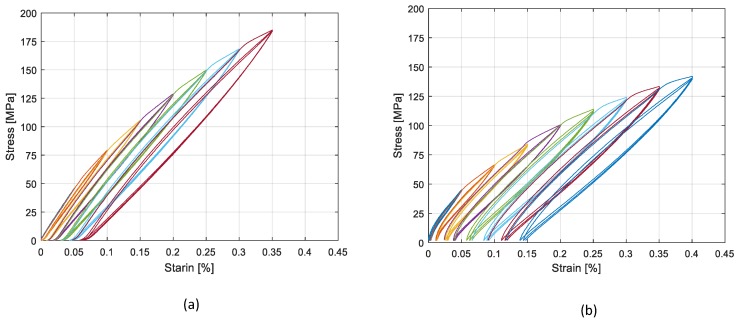
Tensile test results on Sample 5: (**a**) 15${}^{\prime }$ HT; (**b**) 30${}^{\prime }$ HT sheet.

**Figure 13:**
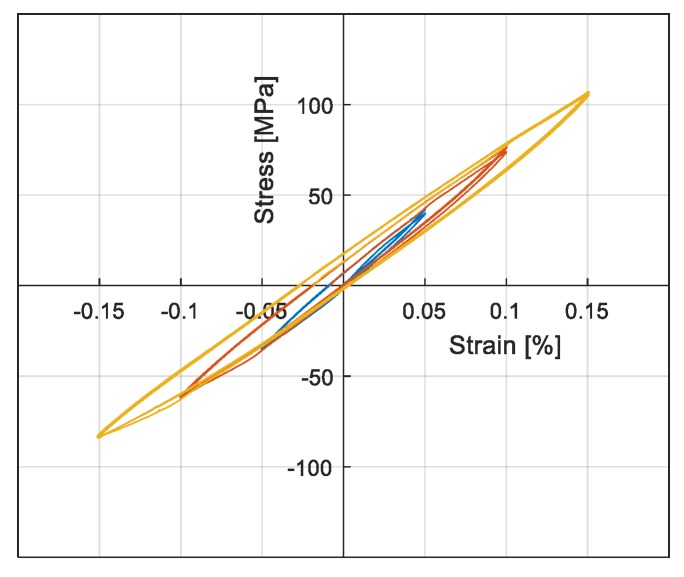
Tensile-compression test results on Sample 3 with 30${}^{\prime }$ HT.

Table 3 shows the tests performed by using experimental techniques. In regard to cyclic tensile tests, cycles were controlled by strain values from 0.05% to 0.4%. For tests with two strain gauges on one side (first setup), the average value of the two strain gauges was considered. The results of tests with different heat treatments are presented in Figure 11 and Figure 12, where consecutive application of cyclic tests with increasing amplitudes are shown with different colors. The stress strain curves were obtained through cyclic tensile tests on Specimen 1 and are presented in Figure 11. As observed in Figure 11a, before the heat treatment the material exhibited a linear elastic behavior under cyclic test conditions, while it exhibited large hysteresis cycles after performing the heat treatment, as presented in Figure 11b. Figure 12a,b, presents the results of cyclic tests performed on sample 5 with a 15${}^{\prime }$ and 30${}^{\prime }$ heat treatment respectively. It is possible to observe that by increasing the heat treatment time, the material in less stiff of material, while at the same time the hysteresis cycles are larger. It was also checked and confirmed that the materials with same heat treatments had similar behaviors. Figure 13 presents the results of the cyclic tensile-compression test in sample 3. Non elliptical hysteresis cycles were observed.

**Figure 14:**
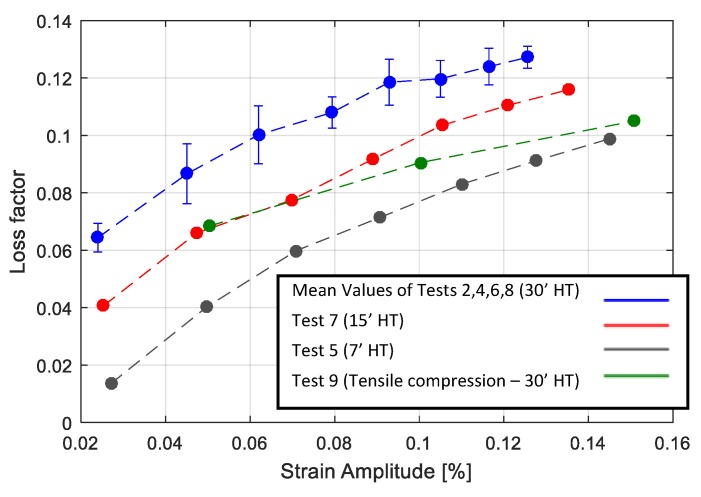
Comparison of the loss factor for Tests 1–9, as reported in Table 3.

In order to obtain a quantitative comparison of the amplitude dependent damping of the material and to investigate the behavior of the heat treatments on damping, with regard to the heat treated samples, the loss factor for hysteresis cycles in each test was calculated as follows:

(2) $$\eta =\;\frac{\Delta U}{2\pi \;U_{max}},$$

where the maximum energy of the system is denoted by $U_{max}$ and the dissipated energy is denoted by ΔU. The loss factors corresponding to the performed tests were calculated, and the results are presented in Figure 14 for all the heat treated samples, where the results of the tensile tests on a 30${}^{\prime }$ heat treatment were averaged.

A high dependency of the loss factor on the deformation amplitude was observed in all cases. It was also observed that a longer heat treatment resulted in a higher loss factor. Consequently, 30${}^{\prime }$ heat treated samples exhibited higher damping values compared to 15${}^{\prime }$ and 7${}^{\prime }$ heat treated samples. With regard to the cyclic tensile compression test results (Figure 13), a more realistic condition in the vibration of structures, lower loss factors were observed compared to the cyclic tensile tests on the specimen with the same heat treatment (30${}^{\prime }$ HT).

Since the high damping capacity of the Cu-based SMAs in the martensite state mainly arose from the movement of various interfaces in the matrix, the increase of the damping in cyclic tensile tests, for samples with a higher heat treatment time, can be attributed to the increase of the amounts of various interfaces in the unit volume. This can be observed in the SEM images, confirming the existence of smaller martensite plates which would give rise to interfaces inside a grain. The other reason could be based on the fact that a longer heat treatment results in a higher grain size, as shown in Figure 5. Actually, the grain size can have two opposite effects on the damping. One is the increase of interfaces, which gives rise to a higher damping; the other is the decrease of the mobility of the interfaces, which leads to the decrease of the damping [14,16]. Consequently, with regards to the investigated material, the existence of smaller martensitic plates inside the grain can be considered as the dominant damping mechanism.

**Figure 15:**
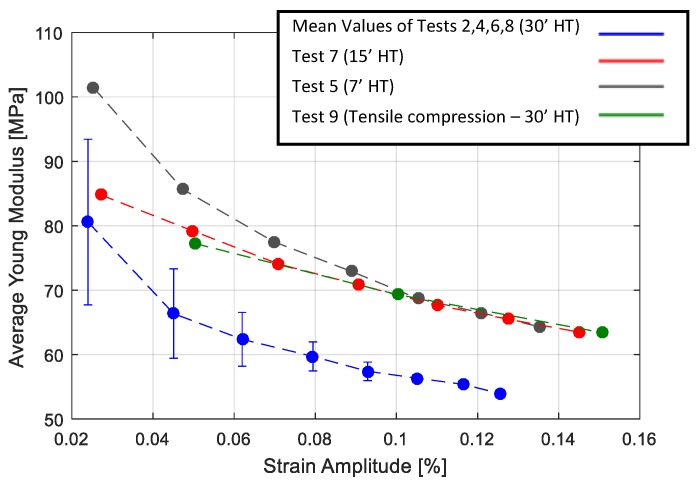
Comparison of the average Young’s modulus for Tests 1–9, as reported in Table 3.

To compare the stiffness of each specimen, the average elastic modulus of each cycle was calculated as:

(3) $$E_{av}=\frac{\sigma_{max}}{\epsilon_{max}},$$

where $\sigma_{max}$ and $\epsilon_{max}$ are the maximum stress and strain of the hysteresis loops. The results are presented in Figure 15. The results of the tensile tests on the 30′ heat treatment are averaged. In all cases, the stiffness of the material decreased when increasing the deformation. It was also possible to observe that a longer heat treatment results in less stiffness of the material in the case of cyclic tensile tests. In the case of cyclic tensile compression tests, the material showed greater stiffness compared to the material with the same heat treatment (30${}^{\prime }$), but tested in the cyclic tensile condition.

## 3. Numerical Model of the Material’s Behavior

The numerical techniques available in the literature to model the material’s damping are usually limited to a linearity assumption. With the aim to model an amplitude dependent damping of SMA numerically, this study applied a phenomenological methodology based on modeling the materials’ hysteresis behaviors. A plot of instantaneous stress versus instantaneous strain, for all values of time during a steady state of forced vibration tests, is referred to as a hysteresis cycle. Indeed, it is a well established experimental approach to classifying and quantifying the internal damping behavior of materials. Metal alloys, and severe high damping alloys, show elliptical hysteresis cycles with a linear damping, while the hysteresis cycles show a more sophisticated shape with a nonlinear damping.

In order to model the nonlinear damping, some studies used viscoelastic material models, such as Kelvin–Voigt, a standard linear solid, or Boltzmann’s models [18]. Although those models can model a high dependency of the total dissipated energy on the strain, drawbacks are given by their sometimes poor accuracy and by their difficulty to identify the model parameters. Few researchers have focused on the use of phenomenological nonlinear damping models. Gottlieb and Habib [19] used a phenomenological nonlinear damping model to understand the large amplitude vibrations of a spherical pendulum.

The composition investigated in this study showed that hysteresis cycles are not elliptical, as observed in Figure 13. Moreover, it showed a non linear damping with respect to the strain, as observed in Figure 14. In order to model this behavior, a phenomenological approach was developed, based on Masing’s rules, to reproduce SMA’s hysteresis loops when they are in a martensitic state. The model did not consider the internal mechanisms in the damping of material. It was developed with the aim to replicate the nonlinear damping of the material investigated at low ranges of strain (below 0.5%). The model was implemented in the user material subroutine of Abaqus FE Code (UMAT).

### 3.1. Introducing the Model

**Figure 16:**
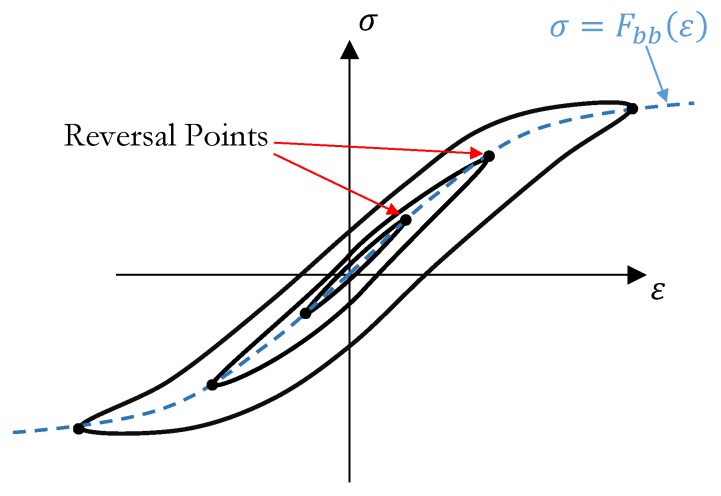
Schematic of the Masing hysteresis cycle.

Masing rules were originally introduced in 1926 [20] and then extended by Karmer [21] in 1966 to four statements:

- 1. For initial loading, the stress strain curve follows the backbone curve (See Figure 16) which can be defined as:
  (4) $$\sigma =F_{bb}\left(\epsilon \right),$$
  where $F_{bb}\left(\epsilon \right)$ is called backbone function.
- 2. If a stress reversal (see Figure 16) occurs at a point defined by ( $\varepsilon_{rev}$, $\sigma_{rev}$ ), the stress strain path will be given by:
  (5) $$\frac{\sigma -\sigma_{rev}}{2}=F_{bb}\left(\frac{\varepsilon -\varepsilon_{rev}}{2}\right).$$
- 3. If the loading curve intersects the backbone curve, it follows the backbone curve until the next stress reversal.
- 4. If an unloading or reloading curve crosses an unloading or reloading curve from the previous cycle, the stress strain curve follows that of the previous cycle.

In order to model the behavior of the investigated material, this study implemented the modified formulation of the second rule suggested by G. Muravskii [22,23], where Equation (3) takes the form:

(6) $$\sigma -\sigma_{rev}=\varphi \left(\varepsilon -\varepsilon_{rev}\right),$$

where φ is the hysteresis function. The hysteresis function implemented in the developed model is presented in Equation (7), where α is considered a parameter which should be tuned on the basis of the material’s behavior. Lower values of α lead to a smaller hysteresis cycle, while higher values would result in bigger hysteresis loops and consequently higher damping. Setting α equal to 2, would regenerate the original Masing rule.

(7) $$\varphi =\alpha F(\frac{\varepsilon -\varepsilon_{rev}}{\alpha }).$$

Rules 2 and 3 were implemented as they were introduced in the original version of the Masing model, whereas Rule 4 was not implemented, since the related condition was not observed in the performed experimental tests.

**Figure 17:**
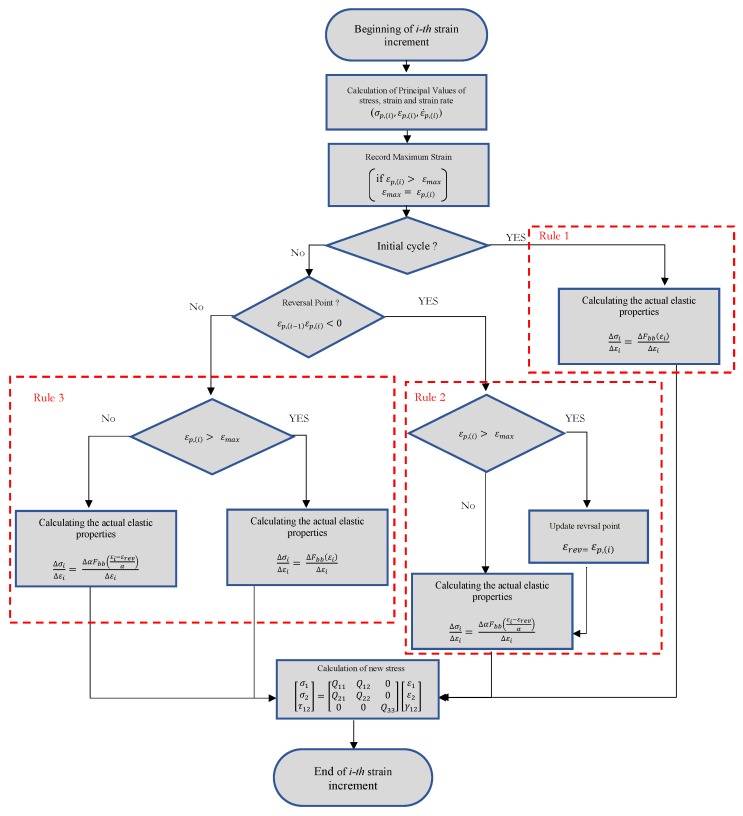
Flowchart of the UMAT code for implementing Masing rules.

Figure 17 shows the flowchart of the developed UMAT. The model requires a backbone curve and parameter α in Equation (7) as inputs to be introduced by the user. The developed model calculates the actual elastic properties for each strain increment, on the basis of the actual principal value of strain ($\varepsilon_{p,(i)}$) and the last reversal strain ($\varepsilon_{rev}$). Reversal points are detected by the sign change of actual and previous principal strains. The principal strain with the highest absolute value was considered. Hooke’s law relating to stress on strains for elastic isotropic materials in plane stress conditions was implemented in the UMAT to calculate the stress at each increment according to Equations (8)–(11).

(8) $$\left[\begin{array}{c} \sigma_{1}\\ \sigma_{2}\\ \tau_{12} \end{array}\right]=\left[\begin{array}{ccc} Q_{11} & Q_{12} & 0\\ Q_{12} & Q_{11} & 0\\ 0 & 0 & Q_{33} \end{array}\right]\left[\begin{array}{c} \varepsilon_{1}\\ \varepsilon_{2}\\ \gamma_{12} \end{array}\right]$$

(9) $$Q_{11}=\frac{E_{\left(i\right)}}{1-\nu_{12}^{2}}$$

(10) $$Q_{12}=\frac{\nu_{12}E_{\left(i\right)}}{1-\nu_{12}^{2}}$$

(11) $$Q_{33}=G_{21}=\frac{E_{\left(i\right)}}{2(1+\nu_{12})}.$$

### 3.2. Characterizing and Validation of the Proposed Model

The results of Test 1 were chosen as reference of the numerical model. The backbone curve related to this test (Figure 11) was fitted and introduced in the numerical model by a 4th order polynomial (Equation (12)). The polynomial constant parameters are presented in Table 4. The same table shows the α value (Equation (7)) tuned based on the experimental material behavior at different maximum strain amplitudes.

The validation of the proposed model was performed through a numerical analysis carried out with Abaqus FE Code. The numerical analysis was based on a plain stress model of the experimental specimen and on the material’s behavior simulated by the UMAT subroutine described in the previous section.

**Figure 18:**
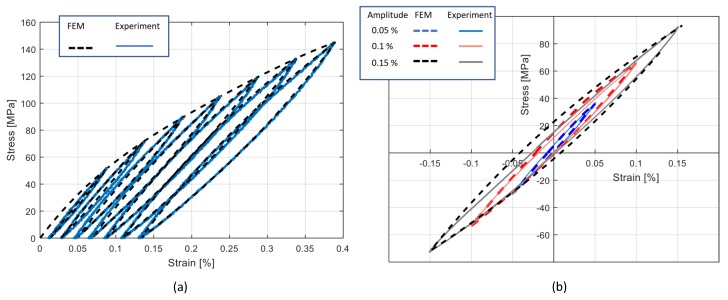
Comparison of the numerical and experimental cycles: (**a**) cyclic tensile Test 1; (**b**) tensile/compression test.

The comparison of hysteresis behavior under experiments—the simulated cyclic tensile and tensile compression tests, are presented respectively in Figure 18a,b. In regard to the cyclic tensile tests, as depicted in Figure 18a, the experimental and numerical diagrams were in good agreement. For each maximum strain amplitude, the experimental cycle was well reproduced. Also in the case of the cyclic tensile compression tests, the experimental and numerical diagrams were in good agreement, as depicted in in Figure 18b. However, at a high amplitude, the numerical diagram was less accurate, and it described a hysteresis cycle with a higher value of the associated dissipated energy.

**Figure 19:**
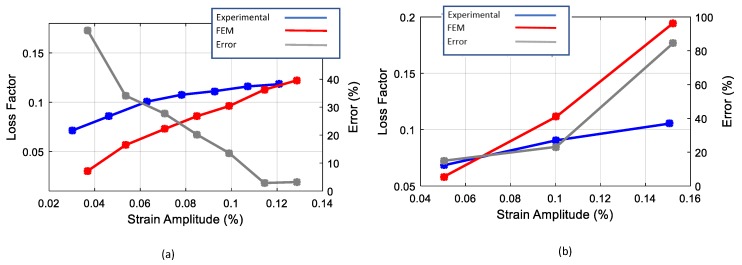
Comparison of the numerical and experimental loss factor: (**a**) cyclic tensile test; (**b**) tensile/compression.

**Figure 20:**
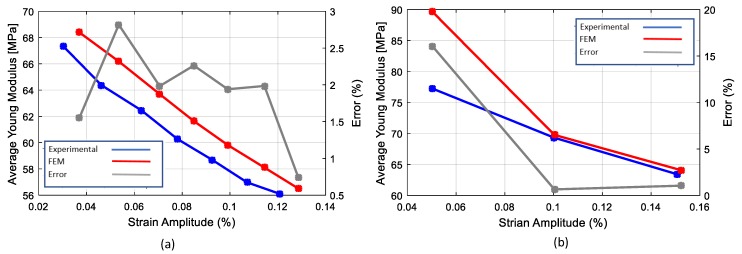
Comparison of the numerical and experimental average Young’s modulus: (**a**) cyclic tensile test; (**b**) tensile/compression.

In both cases, in order to obtain a quantitative comparison, each cycle reproduced was compared with the corresponding experimental one in terms of loss factor and average elastic modulus. The results are presented in Figure 19 and Figure 20 respectively. It was observed that the model was able to reproduce the non-linear damping of material successfully. The comparison of the loss factor in the case of the cyclic tensile tests (Figure 19a), shows that the numerical model is more accurate for larger cycles with errors below 5%. This is in contrast with the case of the cyclic tensile-compression tests where the numerical simulation results in an overestimation of the damping at high strain amplitudes. The comparison of the average Young’s modulus (Figure 20) shows that the numerical model is accurate enough in both the cyclic tensile and the tensile-compression load cases with an average error of 3%.

(12) $$F_{bb}=a\varepsilon^{4}+b\varepsilon^{3}+c\varepsilon^{2}+d\varepsilon +e$$

## 4. Summary and Conclusions

In this study, high damping CuAlMn sheets with martensitic micro-structure at ambient temperature were fabricated and characterized both experimentally and numerically. A Cu-Al-Mn shape memory alloy with a nominal composition of 11.65 wt.% of Al and 3 wt.% of Mn, was cast and hot rolled successfully to the thickness of 0.4–0.3 mm, which is the thickness required for the sheets to be embedded within the SMA/GFRP hybrid composites.

The transformation temperatures, the micro-structure, and the damping of the material, were studied. A martensite-austenite transformation temperature very high with respect to the ambient temperature was observed, and the transformation temperatures did not vary for samples with different heat treatments.

The internal damping of the material at different amplitudes and for different heat treatments was investigated by experimental measurements of the loss factor parameter during cyclic traction/compression tests. A high dependency of the loss factor on the deformation amplitude was observed in all cases. Moreover, it was observed that a longer heat treatment results in a higher loss factor and in less stiffness of the material.

With the aim to model an amplitude dependent damping of SMA numerically, a model of the materials based on hysteresis behavior is proposed. The effects of the heat treatment on model were calibrated and validated for the investigated material. An acceptable agreement between the experimental and numerical results was observed. The accuracy of the model was evaluated with a comparison between the experimental and predicted average Young’s modulus. An average error of 3% was calculated in both the cyclic tensile and the tensile-compression load cases.

## Figures and Tables

**Table 1:** Dimensions and heat treatment details of the rolled samples.

Specimen Number	Initial Thickness (mm)	Final Thickness (mm)	Heat Treatment
1	3.14	0.35	30${}^{\prime }$ at 900 ${}^{{}^{\circ}}$C + WQ
2	4.12	0.35	30${}^{\prime }$ at 900 ${}^{{}^{\circ}}$C + WQ
3	6.28	1.11	30${}^{\prime }$ at 900 ${}^{{}^{\circ}}$C + WQ
			7${}^{\prime }$ at 900 ${}^{{}^{\circ}}$C + WQ
4	7.25	0.42	15${}^{\prime }$ at 900 ${}^{{}^{\circ}}$C + WQ
			30${}^{\prime }$ at 900 ${}^{{}^{\circ}}$C + WQ
5	7.30	0.45	15${}^{\prime }$ at 900 ${}^{{}^{\circ}}$C + WQ
			30${}^{\prime }$ at 900 ${}^{{}^{\circ}}$C + WQ

**Table 2:** Transformation temperatures.

Sample Description	$A_{s}$ (${}^{{}^{\circ}}$C)	$A_{f}$ (${}^{{}^{\circ}}$C)	$M_{s}$ (${}^{{}^{\circ}}$C)	$M_{f}$ (${}^{{}^{\circ}}$C)
As Cast	472	546	215	169
As hot-rolled to 0.7 mm	481	538	229	198
hot rolled and heat treated for 30${}^{\prime }$ at 900 ${}^{{}^{\circ}}$C	484	568	357	163

**Table 3:** Specification of the cyclic tests performed.

Test	Sample	Heat Treatment	Loading Condition	Strain Measurement
1	No. 1	No Heat treatment	Tensile	Strain gauges
2	No. 1	30${}^{\prime }$ at 900 ${}^{{}^{\circ}}$C	Tensile	Strain gauges
3	No. 2	No Heat treatment	Tensile	Extensometer
4	No. 2	30${}^{\prime }$ at 900 ${}^{{}^{\circ}}$C	Tensile	Extensometer
5	No. 4	7${}^{\prime }$ at 900 ${}^{{}^{\circ}}$C	Tensile	Extensometer
6	No. 4	30${}^{\prime }$ at 900 ${}^{{}^{\circ}}$C	Tensile	Extensometer
7	No. 5	15${}^{\prime }$ at 900 ${}^{{}^{\circ}}$C	Tensile	Extensometer
8	No. 5	30${}^{\prime }$ at 900 ${}^{{}^{\circ}}$C	Tensile	Extensometer
9	No. 3	30${}^{\prime }$ at 900 ${}^{{}^{\circ}}$C	Tensile-Compression	Strain gauges

**Table 4:** Parameters used in the model.

Hysteresis Function	Backbone Curve Fitting Line				
α	a	b	c	d	e
2.5	$-5.0823\times 10^{17}$	$5.233\times 10^{15}$	$-2.175\times 10^{13}$	$6.9696\times 10^{10}$	$4.159\times 10^{5}$
